# Clinical validation of the Integrative Vitality Scale: a screening and patient-centered assessment tool for frailty and depressive disorders

**DOI:** 10.3389/fpubh.2026.1788260

**Published:** 2026-03-31

**Authors:** Seok-In Yoon, Hui-Yeong Park, Jiho Pyun, Yerim Jeon, Sun-Yong Chung, Jong Woo Kim

**Affiliations:** 1 Department of Neuropsychiatry, College of Korean Medicine, Kyung Hee University, Seoul, Republic of Korea; 2 Department of Neuropsychiatry in Korean Medicine, Kyung Hee University Medicine Center, Seoul, Republic of Korea; 3 Department of Neuropsychiatry, Kyung Hee University Korean Medicine Hospital at Gangdong, Seoul, Republic of Korea

**Keywords:** Integrative Vitality Scale, subjective vitality, frailty, depressive disorder, healthy ageing, screening, clinical validation, incremental validity

## Abstract

**Background:**

Vitality refers to an organism’s inherent energy that supports adaptive functioning and well-being. Both frailty and depressive disorders are characterized by energy depletion and are commonly associated with fatigue, reduced subjective well-being, and impaired quality of life. Assessing vitality may serve as a complementary approach to identify conditions associated with energy depletion, while offering clinically meaningful information beyond that captured by disorder-specific measures alone. The Integrative Vitality Scale (IVS) measures the physical and psychological dimensions of vitality. This study examined the clinical validity of the IVS in frailty and depressive disorders.

**Methods:**

Data were drawn from two independent South Korean samples: adults aged ≥65 years assessed for frailty (Study 1) and adults aged ≥19 years assessed for depressive disorders (Study 2). Correlation analysis was used to examine the associations between IVS scores and fatigue, subjective well-being, and quality of life. Receiver operating characteristic (ROC) curve analysis was used to evaluate screening performance against structured clinical interview-based reference standards. Hierarchical regression analyses were used to test the incremental explanatory value of the IVS beyond the established measures of frailty and depression.

**Results:**

The IVS total and subscale scores were negatively associated with frailty, depressive symptoms, and fatigue, and positively associated with subjective well-being and quality of life. Physical vitality demonstrated good screening performance, with overall discriminative ability in a similar range to that of a clinician-rated frailty measure. For depressive disorders, the IVS total score showed fair screening performance, with overall discriminative ability in a similar range to that of the widely used self-report depression scale. Hierarchical regression analyses further indicated that the IVS explained additional variance in fatigue, subjective well-being, and quality of life beyond the established measures of frailty and depressive symptom severity, with physical vitality primarily related to fatigue, and psychological vitality being more strongly related to well-being and quality of life.

**Conclusion:**

The IVS demonstrated clinical validity as a self-report screening and assessment tool for conditions characterized by energy depletion. Beyond initial screening, the IVS provides patient-centered information on functional status that is not fully captured by disorder-specific measures, supporting its complementary role in clinical screening contexts. Further validation in representative and community-based samples is required before broader public health applications can be considered.

**Clinical trial registration:**

https://cris.nih.go.kr/cris/search/detailSearch.do?seq=27011&status=5&seq_group=27011&search_page=M, identifier KCT0009372; https://cris.nih.go.kr/cris/search/detailSearch.do?seq=26780&status=5&seq_group=26780&search_page=M, identifier KCT0009263.

## 1 Introduction

Vitality, rooted in the ancient Greek concept of vitalism, refers to the inherent life energy of an organism, which is distinct from the chemical processes of inorganic matter. Traditionally, it has been viewed as a fundamental force driving growth, development, and activity. The concept has also been associated with passion, motivation, and spiritual energy (1). In traditional Oriental medicine, analogous terms such as qi, chi, and ki represent both vital energy and universal principles (2).

With an aging global population, especially in developed nations, the proportion of older adults is projected to reach 16.4% by 2050 (3). In response, the United Nations emphasized “healthy aging” as a key objective within its Sustainable Development Goals. The World Health Organization (WHO) has identified vitality as one of five core competencies essential for achieving healthy aging (4). Consequently, developing tools to assess inherent competencies such as vitality has become a global health priority (5).

Traditionally, vitality has been assessed using physiological indicators, such as heart rate, respiration, body temperature, and blood pressure. Recently, pain and walking speed have been proposed as the fifth and sixth vital signs, respectively (6, 7). Physical performance measures, including grip strength (8) and cognitive function (9), serve as objective indicators. However, a strictly mechanistic approach may overlook the original meaning of vitality as conceptualized by vitalism (2). Vitality is recognized as a subjective, positive experience of bodily energy rooted in individuals’ self-perceptions, rather than purely objective metrics (10). Subjective health assessments have demonstrated predictive validity comparable to objective indicators such as medical history, disability, and medication use for predicting adverse health outcomes, including chronic disease, functional decline, and mortality (11–13). These findings underscore the relevance of subjective vitality as a meaningful indicator of health status and disease progression.

The Integrative Vitality Scale (IVS) is a self-report measure developed to assess subjective vitality and measure multidimensional aspects of vitality, specifically the physical and psychological dimensions (2). Physical vitality is operationally defined as a positive bodily feeling experienced during adequate relaxation. Psychological vitality is a state of intrinsic motivation that involves interest in and concern for life, enjoyment, and active engagement. According to Yoon et al. (2), the IVS significantly predicts symptoms of low energy, such as depression and fatigue, and its predictive validity exceeds that of other existing vitality scales. However, because the scale was initially validated in a non-clinical population, its applicability in clinical settings remains uncertain. To address this limitation, the present study aimed to evaluate the clinical validity of the IVS in populations characterized by energy deficiency, specifically in individuals with depressive disorders or frailty.

Frailty is a vulnerable state in which physiological resources for homeostatic regulation decrease owing to the overall functional decline caused by pathological aging, making it difficult to respond effectively to external stressors (14). A meta-analysis of 240 studies across 62 countries found that the global prevalences of frailty and pre-frailty were 12 and 46%, respectively (15). In Korea, the prevalence rates range from 2.5 to 11.2% for frailty and 32.1 to 42.7% for pre-frailty (16).

Depressive disorder is characterized by a persistently low mood, diminished pleasure or interest, and is often accompanied by fatigue and feelings of worthlessness (17). As of 2021, it remains one of the most prevalent psychological disorders worldwide, affecting nearly 330 million individuals (18) and ranking second globally in disease burden, as measured by years lived with disability, after low back pain (19). Its prevalence increases with age and physical vulnerability (20, 21).

Frailty and depressive disorders share overlapping features, most notably depletion of physical and psychological energy. In traditional East Asian medicine, frailty and depression are attributed to qi deficiency and stagnation, both of which are viewed as conditions of vital energy imbalance and disharmony (22, 23). Consistent with this perspective, fatigue is a predominant complaint among patients diagnosed with frailty or depressive disorders (14, 24), and is associated with poorer subjective well-being, reduced quality of life, and long-term functional impairment (25–27). These findings suggest that the clinical impact of frailty and depression extends beyond diagnostic classification to encompass co-occurring symptoms, emotional well-being, and functional limitations in daily life. Therefore, beyond establishing diagnoses, patient-centered assessment approaches are required to predict a broad range of physical, emotional, and functional outcomes associated with frailty and depressive disorders.

Frailty and depressive disorders share overlapping clinical features, frequently co-occur, and increase the risk of one another (28), highlighting the need for comprehensive and integrated management strategies. However, extensive assessments and interventions may lead to patient fatigue, resistance, and increased healthcare costs (29). For example, frailty and depressive disorders are typically diagnosed through structured clinical interviews based on distinct criteria that require substantial time, labor, and clinical resources. While clinical interviews are foundational for building therapeutic relationships and guiding diagnosis and treatment, their utility is increasingly constrained by time availability in medical settings (30).

To optimize the use of clinical resources required for a definitive diagnosis, effective initial screening tools with adequate accuracy are essential. Moreover, beyond categorical diagnostic classification, assessment measures are needed to capture and predict patient-centered outcomes such as emotional well-being and quality of life, which are not fully explained by disease severity alone. Accordingly, this study aimed to examine the clinical validity of the IVS by evaluating its performance in identifying frailty and depressive disorders using established clinical criteria as reference standards as well as its incremental explanatory value for fatigue, subjective well-being, and quality of life beyond frailty and depressive symptoms in separate analyses.

## 2 Methods

### 2.1 Overview of study design

Studies 1 and 2 were prospective, single-center, cross-sectional studies conducted at a university hospital in Seoul, Republic of Korea. Participants were recruited using convenience sampling from patients visiting a university hospital who responded to recruitment notices. Written informed consent was obtained from all participants prior to eligibility screening, which was conducted to determine study inclusion. A double-blind diagnostic accuracy design was employed. The participants were blinded to their diagnostic classifications based on the reference standard, and the clinicians conducting the structured clinical interviews were blinded to the results of the index test, which was completed independently by the participants. The study design and reporting followed the STARD guidelines (Supplementary material 1).

### 2.2 Study 1: Frailty

#### 2.2.1 Participants and procedure

For Study 1, participants were included if they (i) reported physical discomfort such as weight loss, fatigue, or reduced physical activity and function and (ii) were 65 years of age or older. Participants were excluded if they (i) were unable to walk independently; (ii) had organic brain disorders such as dementia, stroke, epilepsy, or Parkinson’s disease; or (iii) had conditions that made it difficult to conduct interviews and tests in this study.

Following eligibility screening, the participants underwent a structured clinical interview conducted by trained clinicians to assess their frailty status. According to Fried’s criteria (14), frailty is defined as the presence of three or more of the following five conditions: unintentional weight loss, self-reported exhaustion, low physical activity, slow walking speed, or reduced handgrip strength. Pre-frailty was defined as the presence of one or two criteria, whereas individuals with none of these criteria were classified as non-frail. These criteria served as the reference standard for the diagnosis of frailty. Based on clinical interviews, participants diagnosed with frailty or pre-frailty were classified into the clinical group (positive cases of frailty; PF), whereas those not diagnosed were classified into the non-clinical group (negative cases of frailty; NF). Given the importance of early identification and management of frailty as well as the potential clinical utility of the IVS as a screening test, individuals with pre-frailty were included in the clinical group.

**Figure 1:**
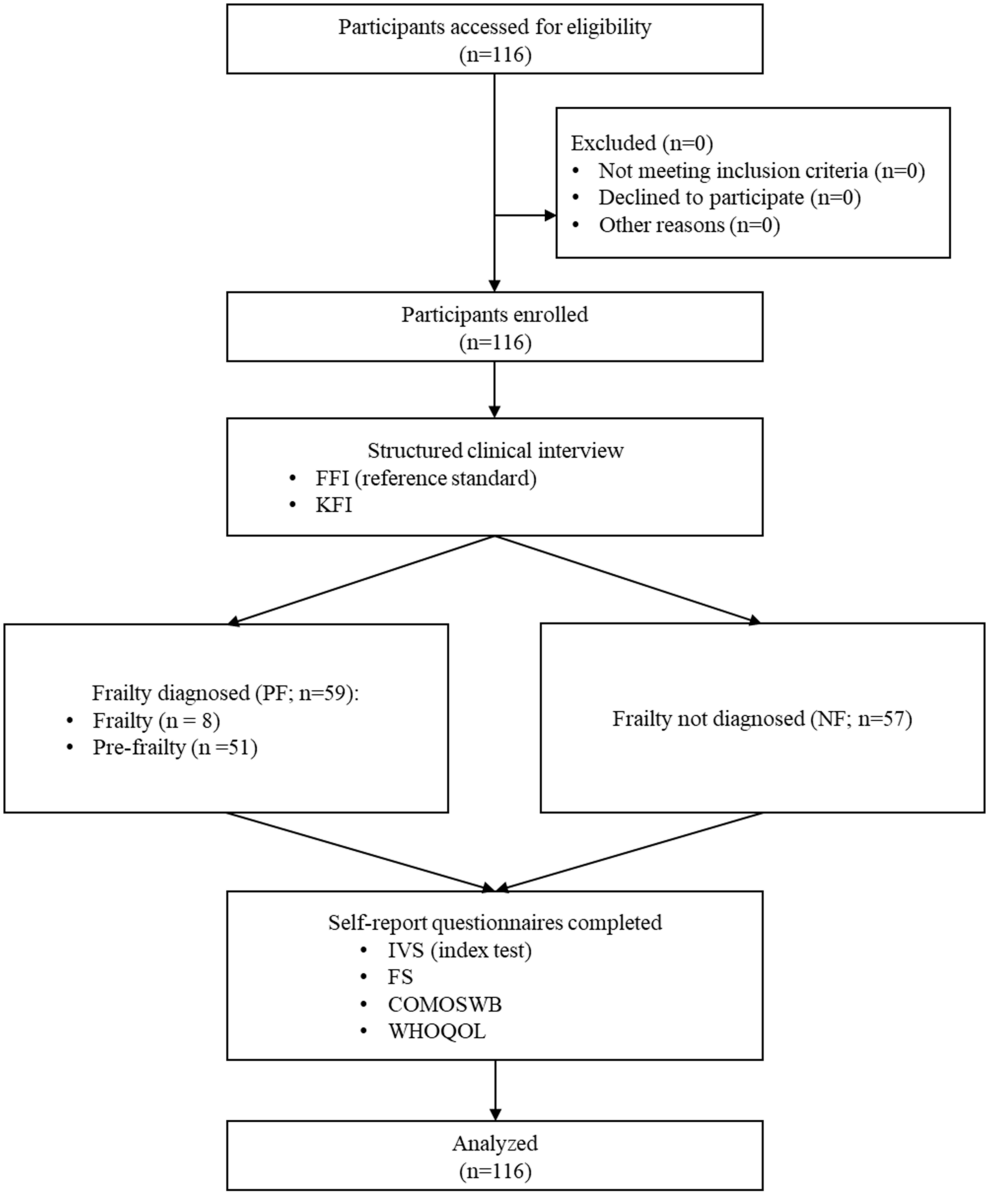
Flow diagram for study 1 examining the clinical validity of the IVS for frailty. COMOSWB, concise measure of subjective well-being; FFI, Fried’s frailty index; FS, fatigue scale; IVS, integrative vitality scales; KFI, Korean frailty index; NF, negative cases of frailty; PF, positive cases of frailty; WHOQOL, Korean version of the World Health Organization quality of life scale abbreviated version.

After the clinical interviews, all participants completed a series of self-report questionnaires, including the IVS. The IVS served as the index test for the identification of frailty. Upon completion of the study procedures, the participants received a compensation of 50,000 KRW. A flow diagram of Study 1 is shown in Figure 1.

The minimum sample size for the receiver operating characteristic (ROC) curve analysis was calculated using MedCalc software (31). With a type I error of 0.01, type II error of 0.05, expected area under the curve (AUC) of 0.725 (considered medium level), and recruitment ratio of 1:1 for positive and negative cases, the required sample size for each group was 53. The data were collected between February 26, 2024, and March 11, 2025. Ultimately, 59 and 57 participants were enrolled in the PF and NF groups, respectively.

#### 2.2.2 Measurements

##### 2.2.2.1 IVS

The IVS was used to assess physical and psychological vitality (2). It is a 22-item self-report measure rated on a 5-point Likert scale comprising two subscales: physical vitality (IVS-P; 11 items) and psychological vitality (emotional–motivational component; IVS-E; 11 items). Total scores ranged from 0 to 44 for each subscale and from 0 to 88 overall, with higher scores indicating higher vitality. In a previous study (2), Cronbach’s alphas were 0.89–0.91 for IVS-P, 0.91–0.94 for IVS-E, and 0.94 for overall. In Study 1, Cronbach’s alpha was 0.88 for the IVS-P, 0.95 for IVS-E, and 0.95 for overall.

##### 2.2.2.2 Fried’s Frailty Index (FFI)

The criteria proposed by Fried et al. (14) were used to diagnose frailty. The criteria consist of five items: unintentional weight loss, self-reported exhaustion, low physical activity, slow walking speed, and poor handgrip strength. Frailty was diagnosed if three or more of the five items were positive and pre-frailty was diagnosed if one or two items were positive. The FFI is a dichotomous scale assessed by a clinician interview, with a total score ranging from 0 to 5.

##### 2.2.2.3 Korean Frailty Index (KFI)

The KFI (32) was used to assess the frailty severity. This is a dichotomous, observer-rated scale administered through clinician interviews. The KFI consists of eight items: number of hospitalizations, subjective health status, medication use, weight loss, depression, incontinence, walking ability, and sensory ability. The total score ranges from 0 to 8.

##### 2.2.2.4 Fatigue Scale (FS)

The FS (33) measures fatigue severity using 14 self-reported items rated on a 4-point Likert scale. The total score ranges from 14 to 56, with a higher score indicating more severe fatigue. In a previous study (34), the Cronbach’s alpha was 0.84; in Study 1, it was 0.91.

##### 2.2.2.5 Concise Measure of Subjective Well-Being (COMOSWB)

The COMOSWB (35) is a 9-item self-report measure that uses a 7-point Likert scale. It comprises of three factors: life satisfaction, positive affect, and negative affect. The total score was calculated as life satisfaction + positive affect – negative affect. Higher scores indicated higher subjective well-being. Cronbach’s alpha was 0.79 in a previous study (35) and 0.89 in Study 1.

##### 2.2.2.6 Korean version of the WHO Quality of Life Scale abbreviated version (WHOQOL)

The WHOQOL, validated in Korean by Min et al. (36), comprises 26 items rated on a 5-point Likert scale. It assesses the following five domains: overall quality of life, physical health, psychological health, social relationships, and environment. Each domain score was calculated by multiplying the mean by 4 (range: 4–20). The total score (range, 20–100) was the sum of the domain scores. Higher scores indicate a better quality of life. Cronbach’s alpha was 0.90 in a previous study (36) and 0.93 in Study 1.

#### 2.2.3 Statistical analysis

##### 2.2.3.1 Preliminary and descriptive analyses

Statistical analyses were performed using SPSS (version 22.0; IBM Corp., Armonk, NY, USA) and R (version 4.1.3). No missing or indeterminate results were observed. Independent t-tests and chi-square tests were performed to compare demographic and clinical characteristics between the groups. Pearson’s correlation analysis was conducted to evaluate the criterion validity of the IVS within the clinical group (PF) and across the entire sample (PF + NF). Correlation coefficients were interpreted according to the guidelines proposed by Mukaka (37), whereby values less than 0.30 indicate very low correlation, 0.30–0.50 low, 0.50–0.70 moderate, 0.70–0.90 high, and greater than 0.90 very high correlation.

##### 2.2.3.2 Primary analyses

Diagnostic accuracy was evaluated by calculating the sensitivity, specificity, and AUC. Because no pre-specified cutoff score for frailty had been established prior to analysis, the optimal cutoff score was determined exploratorily using ROC curve analysis to maximize the sum of sensitivity and specificity. 95% confidence intervals (CIs) for the optimal cutoff values were estimated using bootstrap resampling (2,000 iterations). Sensitivity and specificity were examined at the lower and upper bounds of the CIs. In addition, positive predictive value (PPV), negative predictive value (NPV), positive likelihood ratio (LR+), and negative likelihood ratio (LR−) were calculated to further evaluate the clinical interpretability of each cutoff score. AUC values of 0.50–0.60 were considered failure, 0.60–0.70 poor, 0.70–0.80 fair, 0.80–0.90 good, and 0.90–1.00 excellent. DeLong’s test was used to compare the classification performances of the measures.

A hierarchical regression analysis was conducted to examine the incremental validity of the IVS. For each outcome—fatigue, subjective well-being, and quality of life—sex, age, and the KFI score were entered in Step 1, followed by the inclusion of IVS-P and IVS-E in Step 2. Statistical significance was set at *p* < 0.05.

##### 2.2.3.3 Secondary analyses

Hierarchical binary logistic regression analyses were conducted to examine whether IVS subscales provided incremental explanatory value for diagnostic status beyond established clinical covariates. Diagnostic group (0 = NF, 1 = PF) was entered as the dependent variable. In Step 1, sex, age, and KFI were simultaneously entered as covariates to estimate adjusted odds ratios (*OR*s). In Step 2, IVS-P and IVS-E were added to the model to evaluate their incremental contribution. Model improvement was assessed using the change in model chi-square (*Δχ*^2^) and Nagelkerke *R*^2^. Adjusted *OR*s with 95% CI were reported. Model fit was evaluated using the Hosmer–Lemeshow goodness-of-fit test.

### 2.3 Study 2: depressive disorder

#### 2.3.1 Participants and procedure

Participants were eligible for Study 2 if they (i) reported psychological distress related to depression, such as low mood, reduced motivation, feelings of worthlessness or guilt, and difficulty concentrating, and (ii) were aged 19 years or older. Exclusion criteria were (i) a current or past history of manic or hypomanic episodes; (ii) the presence of hallucinations or delusions; (iii) an organic brain disorder, intellectual disability, or cognitive impairment; and (iv) any conditions that made it difficult to conduct the interviews and assessments used in this study.

Following eligibility screening, participants underwent a structured clinical interview conducted by trained clinicians to determine the presence of major depressive disorder (MDD) (38). According to the DSM-5, MDD is defined as the presence of five or more of the nine core symptoms, such as depressed mood, anhedonia, and changes in appetite, persisting for at least two consecutive weeks, resulting in clinically significant distress or impairment of social or occupational functioning. In addition, the symptoms must not be attributable to the physiological effects of substances or medical conditions and must not be better explained by another psychiatric disorder. These diagnostic criteria served as the reference standard for the diagnosis of depressive disorder. Based on the clinical interview, participants who met the diagnostic criteria for MDD were classified as the clinical group (positive cases of depressive disorder; PDD), whereas those who did not meet the criteria were classified as the non-clinical group (negative cases of depressive disorder; NDD).

**Figure 2:**
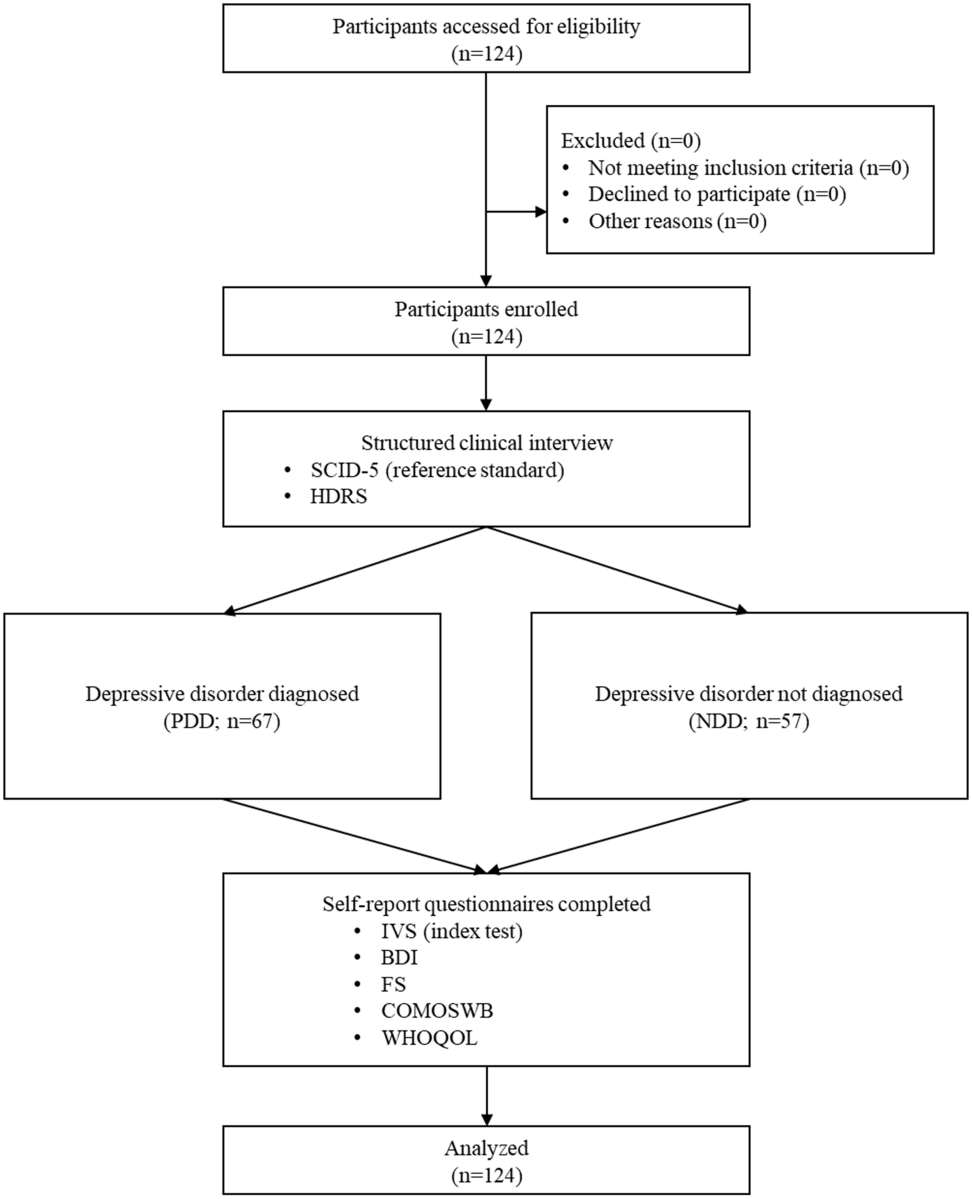
Flow diagram for Study 2 examining the clinical validity of the IVS for depressive disorders. BDI, Korean version of Beck depression inventory; COMOSWB, concise measure of subjective well-being; FS, fatigue scale; HDRS, Korean version of Hamilton depression rating scale; IVS, integrative vitality scales; NDD, negative cases of depressive disorder; PDD, positive cases of depressive disorder; SCID-5, structured clinical interview for DSM-5; WHOQOL, Korean version of the World Health Organization quality of life scale abbreviated version.

After the clinical interviews, all participants completed a series of self-report questionnaires, including the IVS. The IVS served as the index test for identification of depressive disorder. Upon completion of the study procedures, the participants received a compensation of 50,000 KRW. A flow diagram of Study 2 is shown in Figure 2.

The minimum sample size required for ROC curve analysis was calculated using MedCalc software (31). Assuming a type I error of 0.01, type II error of 0.05, expected AUC of 0.725 (considered medium level), and a recruitment ratio of 1:1 for the PDD and NDD groups, the minimum sample size required per group was 53. Data were collected between February 13 and August 28, 2024. Ultimately, 67 and 57 participants were enrolled in the PDD and NDD groups, respectively.

#### 2.3.2 Measurements

##### 2.3.2.1 IVS

The same scales used in Study 1 were utilized in Study 2. Cronbach’s alpha was 0.89 for the IVS-P, 0.96 for the IVS-E, and 0.96 for overall in Study 2.

##### 2.3.2.2 Korean version of the Hamilton Depression Rating Scale (HDRS)

The HDRS (39) was used to assess the severity of depressive symptoms. This 17-item observer-rated scale includes items rated on either a 3-point or 5-point scale, with total scores ranging from 0 to 52. Higher scores indicate more severe depressive symptoms. Clinicians followed a structured interview guide to ensure reliability (40).

##### 2.3.2.3 Beck Depression Inventory (BDI)

The BDI (41) is a 21-item self-report tool used to assess the severity of depressive symptoms. Each item was rated on a 4-point Likert scale, yielding a total score ranging from 0 to 63. Higher scores reflect greater depressive severity. A previous study (41) reported a Cronbach’s alpha of 0.85; in Study 2, it was 0.93.

##### 2.3.2.4 FS

The scale used in Study 1 was also used in Study 2. Cronbach’s alpha was 0.92.

##### 2.3.2.5 COMOSWB

The scale used in Study 1 was also used in Study 2. The overall Cronbach’s alpha was 0.92.

##### 2.3.2.6 WHOQOL

The scale used in Study 1 was also used in Study 2. Cronbach’s alpha was 0.95.

#### 2.3.3 Statistical analysis

##### 2.3.3.1 Preliminary and descriptive analyses

Statistical analyses were performed using SPSS (version 22.0), AMOS (version 22.0; IBM Corp., Armonk, NY, USA), and R (version 4.1.3). No missing or indeterminate results were observed. Independent t-tests and chi-square tests were performed to compare demographic and clinical characteristics between the groups. Pearson’s correlation analysis was conducted to evaluate the criterion validity of the IVS within the clinical group (PDD) and across the entire sample (PDD + NDD). Correlation coefficients were interpreted according to the guidelines proposed by Mukaka (37), whereby values less than 0.30 indicate very low correlation, 0.30–0.50 low, 0.50–0.70 moderate, 0.70–0.90 high, and greater than 0.90 very high correlation.

##### 2.3.3.2 Primary analyses

Diagnostic accuracy was evaluated by calculating the sensitivity, specificity, and AUC. Because no pre-specified cutoff score for depressive disorder had been established prior to the analysis, the optimal cutoff value was determined exploratorily using ROC curve analysis to maximize the sum of sensitivity and specificity. 95% CIs for the optimal cutoff values were estimated using bootstrap resampling (2,000 iterations). Sensitivity and specificity were examined at the lower and upper bounds of the CIs. In addition, PPV, NPV, LR+, and LR − were calculated to further evaluate the clinical interpretability of each cutoff score. AUC values of 0.50–0.60 were considered failure, 0.60–0.70 poor, 0.70–0.80 fair, 0.80–0.90 good, and 0.90–1.00 excellent. DeLong’s test was used to compare the classification performances of the measures.

A hierarchical regression analysis was conducted to examine the incremental validity of the IVS. For each outcome—fatigue, subjective well-being, and quality of life—sex, age, and the BDI score were entered in Step 1, followed by the inclusion of IVS-P and IVS-E in Step 2. Statistical significance was set at *p* < 0.05.

##### 2.3.3.3 Secondary analyses

Hierarchical binary logistic regression analyses were conducted to examine whether IVS subscales provided incremental explanatory value for diagnostic status beyond established clinical covariates. Diagnostic group (0 = NDD, 1 = PDD) was entered as the dependent variable. In Step 1, sex, age, and BDI were simultaneously entered as covariates to estimate adjusted *OR*s. In Step 2, IVS-P and IVS-E were added to the model to evaluate their incremental contribution. Model improvement was assessed using *Δχ*^2^ and Nagelkerke *R*^2^. Adjusted *OR*s with 95% CI were reported. Model fit was evaluated using the Hosmer–Lemeshow goodness-of-fit test.

To further examine the discriminant validity of the IVS relative to FS and BDI, additional measurement model analyses were conducted. Confirmatory factor analyses compared a one-factor model, in which all parcel indicators loaded on a single latent factor, with a three-factor model specifying integrative vitality (IVS), depression, and fatigue as distinct but correlated constructs. Discriminant validity was further evaluated using the Fornell–Larcker criterion based on average variance extracted (AVE) and the heterotrait–monotrait ratio of correlations (HTMT), calculated from observed parcel correlations (42, 43).

## 3 Results

### 3.1 Study 1: Frailty

#### 3.1.1 Demographic and clinical characteristics

Demographic and clinical characteristics of the PF and NF groups were compared. The results are summarized in Table 1. The PF group had significantly higher KFI and FS scores than the NF group, whereas the IVS, COMOSWB, and WHOQOL scores were significantly lower. No significant differences were observed between groups in terms of age or sex.

**Table 1:** Demographic and clinical characteristics of frailty and non-frailty groups.

Measure	Mean ± SD or Rate, % (n)		*t* / *χ*^2^	*p*	*d*
	PF (*n* = 59)	NF (*n* = 57)			
Sex					
Male	23.7 (14)	21.1 (12)	0.119	0.730	0.06
Female	76.3 (45)	78.9 (45)			
Age, y	70.03 ± 4.00	70.30 ± 3.35	0.385	0.701	0.07
IVS	42.00 ± 15.35	57.23 ± 14.21	5.540	<0.001	1.03
IVS-P	19.00 ± 6.81	27.49 ± 7.17	6.542	<0.001	1.22
IVS-E	23.00 ± 9.66	29.74 ± 8.21	4.041	<0.001	0.75
KFI	2.59 ± 1.34	1.14 ± 1.08	−6.423	<0.001	1.19
FS	37.32 ± 6.90	29.82 ± 5.94	−6.262	<0.001	1.16
COMOSWB	12.41 ± 10.72	19.84 ± 9.05	4.042	<0.001	0.75
WHOQOL	58.18 ± 11.19	67.11 ± 9.33	4.660	<0.001	0.87

COMOSWB, concise measure of subjective well-being; FS, fatigue scale; IVS, Integrative vitality scales; IVS-E, psychological vitality subscale; IVS-P, physical vitality subscale; KFI, Korean frailty index; NF, negative cases of frailty; PF, positive cases of frailty; WHOQOL, Korean version of the World Health Organization quality of life scale abbreviated version.

#### 3.1.2 Criterion validity

Pearson’s correlation analyses were performed for both the PF group and the entire sample. The results are summarized in Table 2. In the PF group, the IVS-total and its subscales showed low to moderate negative correlations with FFI, KFI, and FS, and moderate positive correlations with COMOSWB and WHOQOL. In the entire sample, IVS-total and IVS-P showed moderate to high negative correlations with FFI, KFI, and FS and moderate to high positive correlations with COMOSWB and WHOQOL. The IVS-E showed low to moderate negative correlations with FFI, KFI, and FS, and moderate to high positive correlations with COMOSWB and WHOQOL.

**Table 2:** Criterion validity of the IVS for frailty.

Measure	PF (*n* = 59)			Entire sample (*n* = 116)		
	IVS	IVS-P	IVS-E	IVS	IVS-P	IVS-E
FFI	−0.440^***^	−0.468^***^	−0.369^**^	−0.542^***^	−0.586^***^	−0.442^***^
KFI	−0.472^***^	−0.531^***^	−0.375^**^	−0.559^***^	−0.586^***^	−0.471^***^
FS	−0.696^***^	−0.649^***^	−0.648^***^	−0.743^***^	−0.766^***^	−0.636^***^
COMOSWB	0.637^***^	0.499^***^	0.661^***^	0.713^***^	0.621^***^	0.709^***^
WHOQOL	0.678^***^	0.554^***^	0.687^***^	0.700^***^	0.631^***^	0.676^***^

COMOSWB, concise measure of subjective well-being; FFI, Fried’s frailty index; FS, fatigue scale; IVS, Integrative vitality scales; IVS-E, psychological vitality subscale; IVS-P, physical vitality subscale; KFI, Korean Frailty Index; PF, positive cases of frailty; WHOQOL, Korean version of the World Health Organization quality of life scale abbreviated version.

^**^p < 0.01; ^***^p < 0.001.

#### 3.1.3 Classification performance of IVS for frailty

**Figure 3:**
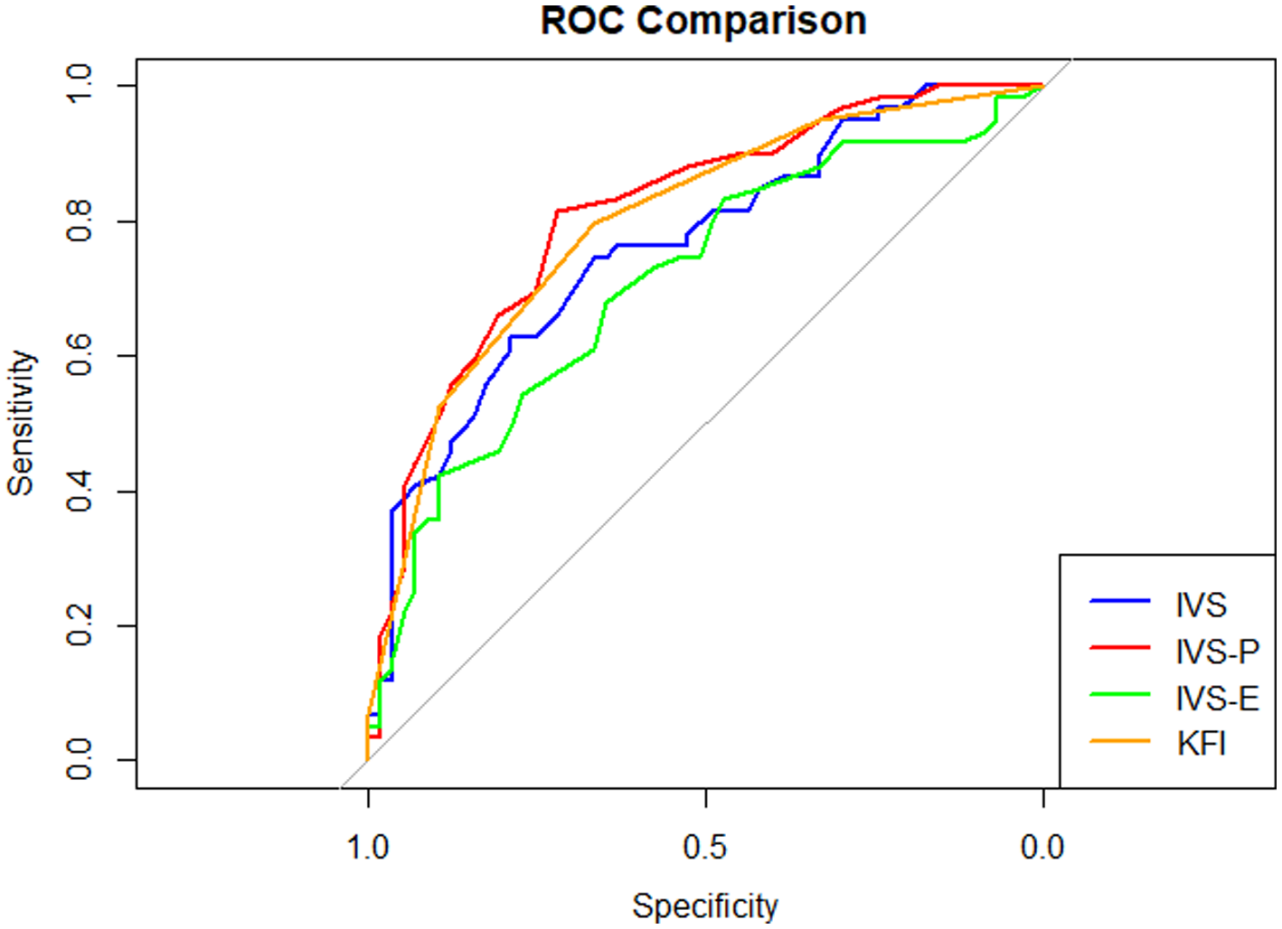
Area under the curve of measurements for diagnosing frailty. Area under the curve values: IVS = 0.762, IVS-P = 0.813, IVS-E = 0.710, KFI = 0.798.

ROC curve analysis was conducted to evaluate the classification performance of the frailty measures (Figure 3). The AUC values were 0.762 (95% CI, 0.676–0.848) for the IVS-total, 0.813 (95% CI, 0.735–0.891) for the IVS-P, 0.710 (95% CI, 0.616–0.805) for the IVS-E, and 0.798 (95% CI, 0.717–0.879) for the KFI. According to DeLong’s test (Supplementary material 2A), the AUC of the IVS-P was significantly higher than those of IVS-total and IVS-E. In addition, the AUC of the IVS-total was significantly higher than that of the IVS-E. The AUC of the KFI did not differ significantly from those of the IVS-total or IVS subscales (*p*s > 0.089).

The optimal cutoff score for the IVS-P for identifying frailty was 24.5 (95% CI, 20.5–25.5), yielding a sensitivity of 0.814 and a specificity of 0.719; at the lower bound (IVS-*p* = 20.5), sensitivity and specificity were 0.559 and 0.877, respectively, whereas at the upper bound (IVS-*p* = 25.5), sensitivity increased to 0.831 and specificity decreased to 0.632. At the optimal cutoff, PPV was 0.750, NPV was 0.789, LR + was 2.90, and LR − was 0.26 (Supplementary material 3).

#### 3.1.4 Incremental validity

Hierarchical regression analyses were conducted to examine the incremental validity of the IVS, beyond the KFI, for fatigue, subjective well-being, and quality of life. The results are summarized in Table 3. After controlling for the KFI, the IVS accounted for a significant proportion of additional variance in all three outcomes.

**Table 3:** Incremental validity of the IVS compared with the KFI.

IV	Step	DV	*B*	*SE*	*β*	*t*	*R* ^2^	*ΔR* ^2^	*F*
FS	Step 1	sex	0.097	1.252	0.005	0.077	0.472	0.472	33.401^***^
		age	−0.137	0.142	−0.068	−0.959			
		KFI	3.566	0.363	0.678	9.837^***^			
	Step 2	sex	−0.232	0.989	−0.013	−0.234	0.678	0.206	46.296^***^
		age	−0.088	0.113	−0.044	−0.783			
		KFI	1.848	0.352	0.351	5.251^***^			
		IVS-P	−0.437	0.082	−0.479	−5.314^***^			
		IVS-E	−0.081	0.065	−0.104	−1.258			
COMOSWB	Step 1	sex	3.129	2.085	0.124	1.501	0.275	0.275	14.134^***^
		age	0.288	0.237	0.100	1.215			
		KFI	−3.780	0.604	−0.506	−6.259^***^			
	Step 2	sex	3.183	1.644	0.126	1.936	0.559	0.284	27.877^***^
		age	0.146	0.188	0.051	0.778			
		KFI	−1.438	0.585	−0.192	−2.457^*^			
		IVS-P	0.147	0.137	0.114	1.077			
		IVS-E	0.589	0.107	0.532	5.484^***^			
WHOQOL	Step 1	sex	3.119	2.234	0.117	1.397	0.259	0.259	13.068^***^
		age	0.403	0.254	0.133	1.587			
		KFI	−3.826	0.647	−0.483	−5.914^***^			
	Step 2	sex	3.295	1.809	0.123	1.822	0.525	0.266	24.333^***^
		age	0.268	0.206	0.088	1.300			
		KFI	−1.231	0.644	−0.155	−1.913			
		IVS-P	0.295	0.150	0.215	1.962			
		IVS-E	0.512	0.118	0.436	4.333^***^			

COMOSWB, concise measure of subjective well-being; DV, dependent variable; FS, fatigue scale; IV, independent variable; IVS, integrative vitality scales; IVS-E, psychological vitality subscale; IVS-P, physical vitality subscale; KFI, Korean frailty index; WHOQOL, Korean version of the World Health Organization quality of life scale abbreviated version.

Standardized regression coefficients (β) indicate the magnitude of individual predictor effects.

ΔR^2^ represents the incremental effect size associated with adding IVS variables.

^*^p < 0.05. ^***^p < 0.001.

Specifically, IVS-P showed a relatively stronger association with fatigue, as reflected by a larger standardized regression coefficient (*β*), whereas IVS-E demonstrated stronger associations with subjective well-being and quality of life. At the model level, the inclusion of IVS variables resulted in moderate increases in explained variance (*ΔR*^2^), indicating that the IVS provides incremental explanatory value beyond the KFI.

### 3.2 Study 2: Depressive disorder

#### 3.2.1 Demographic and clinical characteristics

The demographic and clinical characteristics of the PDD and NDD groups were compared, and the results are presented in Table 4. The PDD group had significantly higher HDRS, BDI, and FS scores than the NDD group, whereas the IVS, COMOSWB, and WHOQOL scores were significantly lower. No significant differences were observed in sex distribution between the groups; however, the PDD group were significantly older than the NDD group. When age was included as a covariate, group differences in the IVS, HDRS, BDI, FS, COMOSWB, and WHOQOL remained statistically significant (*p*s < 0.05).

**Table 4:** Demographic and clinical characteristics of depressive disorder and non-depressive disorder groups.

Measure	Mean ± SD or Rate, % (n)		*t*/*χ*^2^	*p*	*d*
	PDD (*n* = 67)	NDD (*n* = 57)			
Sex					
Male	7.5 (5)	15.8 (9)	2.132	0.144	0.26
Female	92.5 (62)	84.2 (48)			
Age, y	54.58 ± 8.02	46.21 ± 13.35	−4.140	<0.001	0.75
IVS	26.56 ± 11.80	38.99 ± 15.20	5.120	<0.001	0.92
IVS-P	13.55 ± 5.92	18.28 ± 7.20	4.011	<0.001	0.72
IVS-E	13.01 ± 6.65	20.71 ± 8.96	5.479	<0.001	0.99
HDRS	24.10 ± 4.78	14.95 ± 7.04	−8.325	<0.001	1.50
BDI	26.49 ± 7.87	17.58 ± 9.55	−5.610	<0.001	1.01
FS	41.72 ± 5.67	35.75 ± 7.58	−4.888	<0.001	0.88
COMOSWB	2.18 ± 8.48	9.63 ± 10.30	4.419	<0.001	0.80
WHOQOL	48.10 ± 10.03	58.37 ± 12.00	5.192	<0.001	0.94

BDI, Korean version of Beck depression inventory; COMOSWB, concise measure of subjective well-being; FS, Fatigue Scale; HDRS, Korean version of Hamilton depression rating scale; IVS, integrative vitality scales; IVS-E, psychological vitality subscale; IVS-P, physical vitality subscale; NDD, negative cases of depressive disorder; PDD, positive cases of depressive disorder; WHOQOL, Korean version of the World Health Organization quality of life scale abbreviated version.

#### 3.2.2 Criterion validity

Pearson’s correlation analyses were performed for both the PDD group and the entire sample. The results are summarized in Table 5. In the PDD group, the IVS-total and its subscales exhibited low negative correlations with HDRS, moderate to high negative correlations with BDI and FS, and moderate to high positive correlations with COMOSWB and WHOQOL. In the entire sample, the IVS-total and its subscales showed moderate to high negative correlations with HDRS, BDI, and FS and high positive correlations with COMOSWB and WHOQOL.

**Table 5:** Criterion validity of the IVS for depressive disorders.

Measure	PDD (*n* = 67)			Entire sample (*n* = 124)		
	IVS	IVS-P	IVS-E	IVS	IVS-P	IVS-E
HDRS	−0.419^***^	−0.405^***^	−0.382^*^	−0.674^***^	−0.635^***^	−0.642^***^
BDI	−0.630^***^	−0.537^***^	−0.639^***^	−0.753^***^	−0.655^***^	−0.759^***^
FS	−0.705^***^	−0.675^***^	−0.649^***^	−0.790^***^	−0.754^***^	−0.744^***^
COMOSWB	0.755^***^	0.697^***^	0.718^***^	0.802^***^	0.738^***^	0.777^***^
WHOQOL	0.715^***^	0.657^***^	0.682^***^	0.815^***^	0.752^***^	0.789^***^

BDI, Korean version of Beck depression inventory; COMOSWB, concise measure of subjective well-being; FS, fatigue scale; HDRS, Korean version of Hamilton depression rating scale; IVS, integrative vitality scales; IVS-E, psychological vitality subscale; IVS-P, physical vitality subscale; PDD, positive cases of depressive disorder; WHOQOL, Korean version of the World Health Organization quality of life scale abbreviated version.

^*^p < 0.05; ^***^p < 0.001.

#### 3.2.3 Classification performance of IVS for depressive disorder

**Figure 4:**
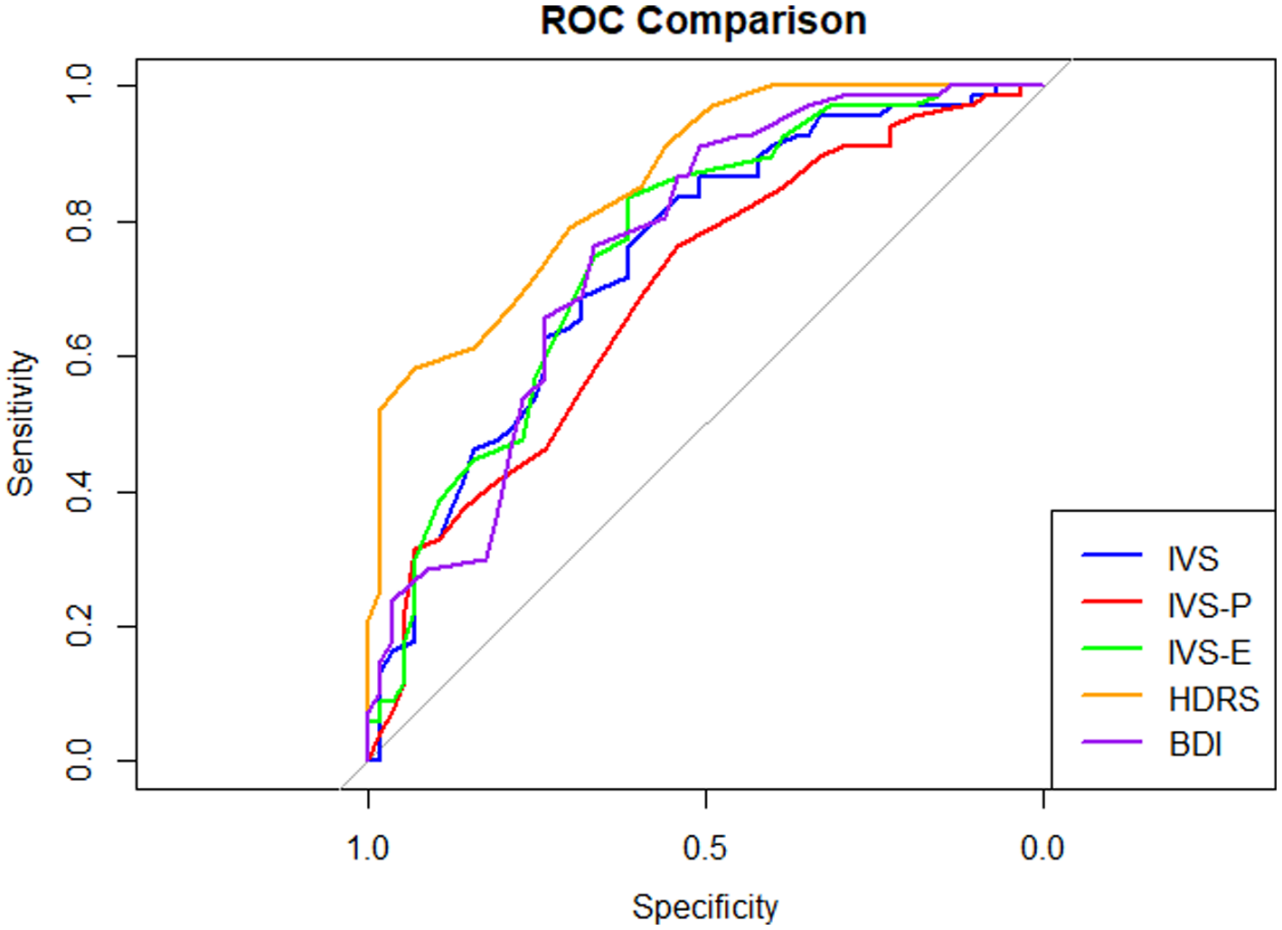
Area under the curve of measurements for diagnosing depressive disorder. Area under the curve values: IVS = 0.743, IVS-P = 0.693, IVS-E = 0.758, HDRS = 0.857, BDI = 0.756.

ROC curve analysis was conducted to evaluate the classification performance of the measures of depressive disorder (Figure 4). The AUC values were 0.743 (95% CI, 0.656–0.830) for the IVS-total, 0.693 (95% CI, 0.600–0.786) for the IVS-P, 0.758 (95% CI, 0.672–0.844) for the IVS-E, 0.857 (95% CI, 0.793–0.920) for the HDRS, and 0.756 (95% CI, 0.670–0.843) for the BDI. According to DeLong’s test (Supplementary material 2B), the AUC of IVS-total was significantly higher than that of IVS-P but did not differ significantly from that of IVS-E. The AUC of the HDRS was significantly higher than those of the IVS-total and all IVS subscales (*p*s < 0.019), whereas the AUC of the BDI did not differ significantly from those of the IVS-total or IVS subscales (*p*s > 0.197).

The optimal cutoff score for the IVS-total for identifying depressive disorder was 36.0 (95% CI, 24.5–42.5), yielding a sensitivity of 0.836 and a specificity of 0.544; at the lower bound (IVS-total = 24.5), sensitivity and specificity were 0.463 and 0.842, respectively, whereas at the upper bound (IVS-total = 42.5), sensitivity increased to 0.910 and specificity decreased to 0.404. At the optimal cutoff, PPV was 0.683, NPV was 0.738, LR + was 1.83, and LR − was 0.30 (Supplementary material 3).

The corresponding cutoff score for the IVS-E was 19.5 (95% CI, 13.5–20.5), with a sensitivity of 0.836 and a specificity of 0.614; at the lower bound (IVS-E = 13.5), sensitivity and specificity were 0.567 and 0.754, respectively, whereas at the upper bound (IVS-E = 20.5), sensitivity increased to 0.866 and specificity decreased to 0.544. At the optimal cutoff, PPV was 0.718, NPV was 0.761, LR + was 2.17, and LR − was 0.27 (Supplementary material 3).

#### 3.2.4 Incremental validity

Hierarchical regression analyses were conducted to examine the incremental validity of the IVS beyond the BDI for fatigue, subjective well-being, and quality of life. The results are summarized in Table 6. After controlling for the BDI, the IVS accounted for a significant proportion of additional variance in all three outcomes.

**Table 6:** Incremental validity of the IVS compared with the BDI.

IV	Step	DV	*B*	*SE*	*β*	*t*	*R* ^2^	*ΔR* ^2^	*F*
FS	Step 1	sex	3.793	1.123	0.167	3.378^***^	0.710	0.710	97.902^***^
		age	−0.045	0.032	−0.072	−1.392			
		BDI	0.621	0.038	0.836	16.292^***^			
	Step 2	sex	2.845	1.011	0.125	2.814^**^	0.776	0.066	81.558^***^
		age	0.010	0.030	0.016	0.332			
		BDI	0.419	0.053	0.563	7.837^***^			
		IVS-P	−0.356	0.079	−0.341	−4.487^***^			
		IVS-E	−0.029	0.070	−0.034	−0.409			
COMOSWB	Step 1	sex	−1.477	1.708	−0.047	−0.865	0.652	0.652	74.856^***^
		age	0.068	0.049	0.078	1.381			
		BDI	−0.849	0.058	−0.822	−14.631^***^			
	Step 2	sex	−0.242	1.524	−0.008	−0.159	0.735	0.084	65.597^***^
		age	−0.008	0.045	−0.009	−0.166			
		BDI	−0.481	0.081	−0.466	−5.972^***^			
		IVS-P	0.380	0.120	0.262	3.175^**^			
		IVS-E	0.247	0.106	0.213	2.336^**^			
WHOQOL	Step 1	sex	−1.520	1.995	−0.040	−0.762	0.672	0.672	81.883^***^
		age	0.015	0.057	0.014	0.262			
		BDI	−1.019	0.068	−0.821	−15.038^***^			
	Step 2	sex	0.147	1.714	0.004	0.086	0.769	0.097	78.405^***^
		age	−0.085	0.051	−0.082	−1.672			
		BDI	−0.550	0.091	−0.443	−6.076^***^			
		IVS-P	0.535	0.135	0.307	3.976^***^			
		IVS-E	0.277	0.119	0.199	2.328^*^			

BDI, Korean version of beck depression inventory; COMOSWB, concise measure of subjective well-being; DV, dependent variable; FS, fatigue scale; IV, independent variable; IVS, integrative vitality scales; IVS-E, psychological vitality subscale; IVS-P, physical vitality subscale; WHOQOL, Korean version of the World Health Organization quality of life scale abbreviated version.

Standardized regression coefficients (β) indicate the magnitude of individual predictor effects.

ΔR^2^ represents the incremental effect size associated with adding IVS variables.

^*^p < 0.05; ^**^p < 0.01; ^***^p < 0.001.

Specifically, IVS-P demonstrated relatively stronger associations across all outcomes, showing a significant negative association with fatigue and significant positive associations with subjective well-being and quality of life. In contrast, IVS-E did not show a significant association with fatigue but exhibited significant positive associations with subjective well-being and quality of life. At the model level, the inclusion of IVS variables resulted in small-to-moderate increases in explained variance, indicating that the IVS provides incremental explanatory value beyond the BDI.

### 3.3 Additional analyses

#### 3.3.1 Adjusted associations with diagnostic status

To complement ROC-based classification analyses, hierarchical logistic regression analyses were conducted to estimate adjusted *OR*s for diagnostic status. All models demonstrated acceptable fit based on the Hosmer–Lemeshow goodness-of-fit test (*p*s > 0.05).

In Study 1, models were fitted in a stepwise manner. In Step 1, the model including sex, age, and KFI was statistically significant (*χ*^2^ = 36.533, df = 3, *p* < 0.001), explaining 36.0% of the variance in frailty status. KFI was independently associated with diagnostic status (adjusted *OR* = 2.756, 95% CI: 1.835–4.139, *p* < 0.001), whereas sex and age were not. In Step 2, the addition of IVS-P and IVS-E resulted in a significant improvement in model fit (*Δχ*^2^ = 13.358, df = 2, *p* = 0.001), increasing the explained variance to 46.6%. In the adjusted model, KFI remained independently associated with diagnostic status (adjusted *OR* = 2.019, 95% CI: 1.284–3.175, *p* = 0.002). IVS-P showed a significant inverse association with diagnostic status (adjusted *OR* = 0.853, 95% CI: 0.771–0.944, *p* = 0.002), whereas IVS-E was not significant.

In Study 2, models were fitted in a stepwise manner. In Step 1, the model including sex, age, and BDI was statistically significant (*χ*^2^ = 41.176, df = 3, *p* < 0.001), explaining 37.8% of the variance in depressive disorder status. Age (adjusted *OR* = 1.070, 95% CI = 1.024–1.118, *p* = 0.003) and BDI (adjusted *OR* = 1.122, 95% CI = 1.062–1.185, *p* < 0.001) were independently associated with diagnostic status, whereas sex was not. In Step 2, the addition of IVS-P and IVS-E resulted in a significant improvement in model fit (*Δχ*^2^ = 6.739, df = 2, *p* = 0.034), increasing the explained variance to 42.8%. In the adjusted model, age remained independently associated with diagnostic status (adjusted *OR* = 1.086, 95% CI = 1.034–1.140, *p* = 0.001). IVS-E showed a marginal inverse association with diagnostic status (adjusted *OR* = 0.901, 95% CI = 0.810–1.003, *p* = 0.058), whereas IVS-P was not significant. Notably, BDI was no longer independently associated with diagnosis after inclusion of IVS subscales.

#### 3.3.2 Discriminant validity

Given the conceptual overlap between vitality, fatigue, and depressive symptoms, and the high correlations observed between the IVS, FS, and BDI in Study 2, there was a concern regarding potential construct redundancy. Therefore, additional analyses were performed using the Study 2 sample to evaluate the discriminant validity of the IVS.

The three-factor model demonstrated a substantially better fit to the data than the one-factor model, indicating that IVS, FS, and BDI are better represented as distinct latent constructs rather than a single undifferentiated factor (see Supplementary material 4). According to the Fornell–Larcker criterion, the AVE for the IVS (AVE = 0.781) exceeded the squared correlations with both FS (*r*^2^ = 0.643) and BDI (*r*^2^ = 0.655), indicating that IVS was not empirically reducible to either measure. Consistent with this interpretation, HTMT values were 0.84 for IVS–FS and 0.80 for IVS–BDI, both below the recommended threshold of 0.85 (43), providing further support for discriminant validity.

## 4 Discussion

This study examined the clinical validity of the IVS as an initial screening and assessment tool for conditions characterized by reduced physical and emotional energy, focusing on frailty and depressive disorders. The findings indicate that the IVS may facilitate a more efficient allocation of limited clinical resources by supporting early identification while also capturing patient-centered outcomes beyond categorical diagnostic labels, including emotional well-being and quality of life. In this regard, the IVS appears to have potential utility as a clinically meaningful measure in clinical screening contexts.

Importantly, the screening accuracy of the IVS varied according to the relative contribution of the total score and its subscales, suggesting that different vitality dimensions may be differentially relevant depending on the target condition. In the context of frailty, the IVS-P demonstrated superior performance compared to the IVS-total and IVS-E. Frailty has been conceptualized as a state of reduced physiological capacity to cope with stress (14) and is associated with impaired autonomic regulation, particularly reduced parasympathetic activity (44). Such dysregulation may contribute to impaired relaxation and decreased physical vitality, providing a plausible explanation for the stronger performance of the IVS-P. Accordingly, the IVS-P appears to offer a favorable balance between sensitivity and specificity, supporting its suitability as a first-line screening measure for frailty in older adults.

In contrast, for depressive disorders, the IVS-total performed better than the IVS-P and did not differ significantly from the IVS-E. Depressive disorders involve emotional disturbances and impairments across multiple functional domains including fatigue, sleep, and appetite (17). An exclusive focus on the emotional dimension of vitality may overlook individuals whose depressive symptoms are primarily expressed through somatic complaints (45). From this perspective, an initial screening approach based on the IVS-total, integrating both physical and psychological vitality, may be more appropriate for identifying individuals at risk of depressive disorders.

From a screening perspective, the IVS-total demonstrated high sensitivity but relatively modest specificity for depressive disorders. This pattern reflects a trade-off favoring sensitivity to minimize false-negative cases at the expense of specificity, which is appropriate for an initial screening context where the primary goal is to avoid missing individuals who may require further clinical evaluation. Notably, the IVS-E showed relatively higher specificity, PPV, and LR + compared to the IVS-total, suggesting that it may provide added value as a complementary measure following initial screening to support more refined clinical judgment regarding depressive disorder risk.

Importantly, the hierarchical logistic regression analyses further refined the interpretation of these screening results by examining adjusted associations beyond overall classification performance. The domain-specific pattern observed across frailty and depressive disorder contexts suggests that different vitality dimensions are preferentially aligned with distinct clinical processes. Physical vitality appears to reflect functional vulnerability relevant to frailty, whereas psychological vitality is more closely linked to affective diagnostic processes. The attenuation of conventional indicators after inclusion of IVS subscales indicates that vitality-based dimensions capture clinically meaningful variance that is not fully accounted for by symptom severity alone. This finding supports the role of the IVS as a complementary framework that enhances clinical interpretability rather than a stand-alone replacement for established instruments.

Building on these domain-specific patterns, ROC analyses further translated the observed screening performance into clinically interpretable cutoff values. Exploratory thresholds of an IVS-P score of ≤ 24.5 (95% CI, 20.5–25.5) were suggested for identifying frailty in individuals aged 65 years or older, whereas an IVS-total score of ≤ 36 (95% CI, 24.5–42.5) was indicated for identifying depressive disorders in adults aged 19 years or older. In addition, the IVS-E demonstrated relatively higher specificity, suggesting that an IVS-E cutoff (≤ 19.5; 95% CI: 13.5–20.5) may provide complementary value for refining clinical judgment following initial screening for depressive disorders, rather than serving as a stand-alone threshold.

The CIs for the cutoff values showed a consistent pattern, with lower-bound thresholds favoring higher specificity and upper-bound thresholds favoring higher sensitivity. This suggests that cutoff selection may be flexibly tailored to clinical context: higher thresholds may be preferable for initial screening prioritizing early case detection, whereas lower thresholds may be more suitable for supporting subsequent diagnostic decision-making where greater specificity is required. These findings underscore the context-dependent clinical utility of the IVS, IVS-P, and IVS-E across different stages of assessment.

From the perspective of initial clinical screening, these findings highlight potential practical advantages of the IVS over established clinician-rated and self-report instruments. For frailty, the AUC of the IVS-P was comparable to that of the clinician-rated KFI, suggesting that the IVS may enable a more efficient use of clinical resources by providing similar classification performance through a self-report format. For depressive disorders, the IVS-total demonstrated overall discriminative ability within a similar range to that of the BDI, a widely used self-report measure for depression screening (46). Although the AUC of the IVS-total was lower than that of the clinician-rated HDRS, its sensitivity of 0.836, based on an exploratory cutoff score of 36, supports its utility as a screening tool for identifying individuals who may require further clinical assessment. Taken together, these findings indicate that the IVS may offer a pragmatic balance between screening accuracy and feasibility, particularly in settings where clinician-administered assessments are resource intensive.

Beyond its role as a screening tool, the IVS provides clinically meaningful information that extends beyond categorical diagnoses. Hierarchical regression analyses showed that the IVS explained additional variance in fatigue, subjective well-being, and quality of life, even after controlling for the KFI and BDI scores. These findings indicate that individuals with the same diagnostic status may differ substantially in their symptom burden and functional outcomes, as reflected by distinct IVS subscale patterns. Specifically, the IVS-P was consistently associated with fatigue, highlighting its relevance to energy depletion and somatic exhaustion. In contrast, the IVS-E was more strongly associated with subjective well-being and quality of life, suggesting that the emotional dimension of vitality is more closely related to adaptive functioning in daily life.

The vitality construct assessed by the IVS inevitably overlaps with established measures of vitality, such as the SF-36 Vitality subscale, which primarily reflects subjective energy and fatigue. However, the IVS was designed to capture a broader, multidimensional vitality state encompassing both physical and emotional dimensions. Consistent with this conceptual distinction, a previous study has demonstrated that the IVS provides incremental validity beyond the SF-36 Vitality subscale in predicting health-related outcomes (2). In the present study, additional discriminant validity analyses further indicated that, despite substantial correlations with fatigue and depressive symptom measures, the IVS represents an empirically distinguishable construct. Although the SF-36 Vitality subscale was not included in this study, future research directly comparing these instruments within the same sample would help further clarify their overlap and complementary roles.

### 4.1 Limitations

First, this study did not fully capture the heterogeneity of clinical populations encountered in hospital settings. Studies 1 and 2 examined frailty and depressive disorders separately, and did not account for the presence of comorbid conditions. Frailty and depression frequently co-occur, and are commonly comorbid with a range of other physical and mental disorders (28, 47, 48). Individuals with multiple comorbidities may experience poorer outcomes across various health and well-being indicators, including vitality, than those with a single diagnosis. Future studies should concurrently assess frailty, depressive disorders, and other highly comorbid conditions to evaluate the feasibility of simultaneous screening and to further examine the discriminative utility of the IVS.

Second, the cutoff scores for the IVS in identifying depressive disorders may vary across age groups. Although depression affects individuals throughout their lifespan, the optimal thresholds for identifying depressive disorders may differ between younger and older adults. Previous studies have reported age-related differences in depression severity, including a reverse U-shaped pattern with corresponding variations in diagnostic cutoff scores (49). Similarly, prior research has shown a U-shaped distribution of IVS scores across the lifespan, with lower vitality observed in middle-aged adults than in older adults (2). In this study, the age distribution was skewed toward middle-aged participants, which may have led to an underestimation of the optimal IVS cutoff scores for older adults. Future research should establish age-specific thresholds to improve the screening accuracy across age groups.

Third, we did not examine symptom severity across different IVS score ranges. Although the proposed cutoff scores indicate the likelihood of frailty or depressive disorders, they do not differentiate symptom severity levels (e.g., mild, moderate, or severe). Exploratory analyses suggested that individuals with frailty had substantially lower IVS-P scores than those with pre-frailty (frailty = 12.38 ± 4.50; pre-frailty = 20.04 ± 6.54; *p* < 0.001; *d* = 1.21), indicating the potential utility of IVS scores in reflecting severity. However, the sample size was insufficient to derive severity-specific thresholds. Larger studies are needed to determine whether IVS scores can reliably distinguish the levels of symptom severity.

Fourth, the cross-sectional design of this study precludes causal or predictive inferences regarding the relationship between vitality and disease onset. Accordingly, the present findings should be interpreted as evidence of concurrent associations between vitality and health-related outcomes rather than as indications of prospective effects. Although prior studies have suggested that subjective vitality may be associated with disease incidence and mortality (12, 13), longitudinal data are required to clarify temporal ordering and causal pathways. Future studies employing longitudinal designs should examine whether baseline vitality prospectively relates to subsequent changes in physical and mental health, thereby determining whether vitality functions as a predictive or potentially protective construct over time.

Fifth, this study was conducted in a single East Asian country, which may limit the generalizability of the findings to other cultural and ethnic contexts. The IVS was developed with conceptual grounding in East Asian health-promoting traditions, particularly principles emphasizing the balance of yin and yang and the harmonious circulation of qi, which informed the distinction between physical and psychological vitality. In addition, the operationalization of physical vitality in the IVS draws in part on phenomenological descriptions of bodily experiences in relaxed states, which are prominently articulated in traditional East Asian practice (e.g., meditation and qigong). These elements may reflect culturally shaped modes of experiencing and expressing vitality.

At the same time, several dimensions of the IVS are likely to reflect more culturally universal processes. Physical vitality, as reflected in subjective experiences of relaxation and bodily regulation, aligns with well-established somatic mechanisms associated with parasympathetic activity observed across cultures. Similarly, psychological vitality is operationalized based on motivational and psychological frameworks, such as self-determination theory, which conceptualizes basic psychological needs as fundamental aspects of human functioning across cultural contexts. Nevertheless, cultural differences may influence item interpretation, response patterns, and optimal cutoff thresholds. Therefore, replication studies in culturally and ethnically diverse populations, including Western samples, are warranted to evaluate measurement invariance and to further establish the external validity of the IVS.

Despite these limitations, this study represents an important initial effort to establish the clinical validity of the IVS for conditions associated with reduced physical and emotional vitality. Future research should further evaluate the classification performance of the IVS in the context of pathological aging conditions such as mild cognitive impairment and sarcopenia. Such investigations may extend the clinical applicability of the IVS as a multidimensional assessment tool that encompasses the physical, emotional, and cognitive aspects of pathological aging. In the context of the rapid aging of the global population, the IVS may serve as a valuable subjective measure for assessing and monitoring healthy aging.

## 5 Conclusion

First, the IVS demonstrates clinical validity as an initial screening tool for frailty and depressive disorders, showing overall discriminative ability within a similar range to established self-report and clinician-rated instruments, while offering advantages in feasibility and clinical resource efficiency.

Second, the differential findings across the IVS subscales indicate that physical vitality is more closely associated with fatigue and energy depletion, whereas psychological vitality is more strongly related to subjective well-being and quality of life. This suggests that the IVS captures meaningful heterogeneity in patient functioning that is not fully explained by diagnostic status or symptom severity alone.

Third, the incremental explanatory value of the IVS for fatigue, well-being, and quality of life supports its role as a patient-centered assessment tool that complements traditional diagnostic frameworks by providing a more nuanced understanding of how patients function in daily life.

Taken together, these findings support the clinical utility of the IVS in screening contexts. Its extension to broader public health applications and longitudinal monitoring will require further validation in diverse populations.

## Data Availability

The raw data supporting the conclusions of this article will be made available by the authors, without undue reservation.
