# Supplementary material for: Clinical validation of the Integrative Vitality Scale: a screening and patient-centered assessment tool for frailty and depressive disorders
Source: Front Public Health. 2026 Mar 31;14:1788260. doi: 10.3389/fpubh.2026.1788260 (PMC13076108; doi:10.3389/fpubh.2026.1788260)
Supplement: Supplementary file 2 [file Table_2.docx]

Supplementary Material 2.

| **Supplementary Material 2A.** Pairwise comparisons of AUC values for frailty identification using DeLong’s test | | | | | |
| --- | --- | --- | --- | --- | --- |
| Comparison Type | Comparison | *Δ*AUC | *Z* | *p* | 95% CI for *Δ*AUC |
| Within-IVS | IVS vs. IVS-P | −0.051 | −2.032 | 0.042 | −0.100 to −0.002 |
|  | IVS vs. IVS-E | 0.052 | 2.892 | 0.004 | 0.017 to 0.086 |
|  | IVS-P vs. IVS-E | 0.103 | 2.526 | 0.012 | 0.023 to 0.182 |
| IVS vs. Comparator | IVS vs. KFI | −0.036 | −0.779 | 0.436 | −0.127 to 0.055 |
|  | IVS-P vs. KFI | 0.015 | 0.328 | 0.743 | −0.074 to 0.104 |
|  | IVS-E vs. KFI | −0.088 | −1.700 | 0.089 | −0.188 to 0.013 |
| *Note.* AUC = area under the curve; IVS = Integrative Vitality Scales; IVS-E = psychological vitality subscale; IVS-P = physical vitality subscale; KFI = Korean Frailty Index. | | | | | |

| **Supplementary Material 2B.** Pairwise comparisons of AUC values for depressive disorder identification using DeLong’s test | | | | | |
| --- | --- | --- | --- | --- | --- |
| Comparison Type | Comparison | *Δ*AUC | *Z* | *p* | 95% CI for *Δ*AUC |
| Within-IVS | IVS vs. IVS-P | 0.050 | 2.159 | 0.031 | 0.005 to 0.096 |
|  | IVS vs. IVS-E | −0.015 | −1.101 | 0.267 | −0.041 to 0.011 |
|  | IVS-P vs. IVS-E | −0.065 | −1.873 | 0.061 | −0.133 to 0.003 |
| IVS vs. Comparator 1 | IVS vs. HDRS | −0.114 | −2.735 | 0.006 | −0.195 to −0.032 |
|  | IVS-P vs. HDRS | −0.164 | −3.623 | <0.001 | −0.252 to −0.075 |
|  | IVS-E vs. HDRS | −0.099 | −2.361 | 0.018 | −0.180 to −0.017 |
| IVS vs. Comparator 2 | IVS vs. BDI | −0.013 | −0.329 | 0.742 | −0.091 to 0.065 |
|  | IVS-P vs. BDI | −0.063 | −1.291 | 0.197 | −0.159 to 0.033 |
|  | IVS-E vs. BDI | 0.002 | 0.048 | 0.962 | −0.074 to 0.077 |
| *Note.* AUC = area under the curve; BDI = Korean version of Beck Depression Inventory; HDRS = Korean version of Hamilton Depression Rating Scale; IVS = Integrative Vitality Scales; IVS-E = psychological vitality subscale; IVS-P = physical vitality subscale. | | | | | |
