# Supplementary material for: Clinical validation of the Integrative Vitality Scale: a screening and patient-centered assessment tool for frailty and depressive disorders
Source: Front Public Health. 2026 Mar 31;14:1788260. doi: 10.3389/fpubh.2026.1788260 (PMC13076108; doi:10.3389/fpubh.2026.1788260)
Supplement: Supplementary file 3 [file Table_3.docx]

Supplementary Material 3. Cross-tabulation of IVS cut-off scores and reference standard diagnoses

|  | | | | | |
| --- | --- | --- | --- | --- | --- |
| Classification  task | Index test | | Reference standard | | |
|  |  |  | Positive case | Negative case | Total |
| PF vs. NF | IVS | ≤ 47.5 point | 37 (62.7) | 12 (21.1) | 49 (42.2) |
|  |  | > 47.5 point | 22 (37.3) | 45 (78.9) | 67 (57.8) |
|  |  | Total | 59 (100) | 57 (100) | 116 (100) |
|  | IVS-P | ≤ 24.5 point | 48 (81.4) | 16 (28.1) | 64 (55.2) |
|  |  | > 24.5 point | 11 (18.6) | 41 (71.9) | 52 (44.8) |
|  |  | Total | 59 (100) | 57 (100) | 116 (100) |
|  | IVS-E | ≤ 26.5 point | 40 (67.8) | 20 (35.1) | 60 (51.7) |
|  |  | > 26.5 point | 19 (32.2) | 37 (64.9) | 56 (48.3) |
|  |  | Total | 59 (100) | 57 (100) | 116 (100) |
| PDD vs. NDD | IVS | ≤ 36.0 point | 56 (83.6) | 26 (45.6) | 82 (66.1) |
|  |  | > 36.0 point | 11 (16.4) | 31 (54.4) | 42 (33.9) |
|  |  | Total | 67 (100) | 57 (100) | 124 (100) |
|  | IVS-P | ≤ 16.5 point | 51 (76.1) | 26 (45.6) | 77 (62.1) |
|  |  | > 16.5 point | 16 (23.9) | 31 (54.4) | 47 (37.9) |
|  |  | Total | 67 (100) | 57 (100) | 124 (100) |
|  | IVS-E | ≤ 19.5 point | 56 (83.6) | 22 (38.6) | 78 (62.9) |
|  |  | > 19.5 point | 11 (16.4) | 35 (61.4) | 46 (37.1) |
|  |  | Total | 67 (100) | 57 (100) | 124 (100) |
| *Note.* IVS = Integrative Vitality Scales; IVS-E = psychological vitality subscale; IVS-P = physical vitality subscale; NDD = negative cases of depressive disorder; NF = negative cases of frailty; PDD = positive cases of depressive disorder; PF = positive cases of frailty.  Values are presented as number (%) | | | | | |
