# Supplementary material for: Clinical validation of the Integrative Vitality Scale: a screening and patient-centered assessment tool for frailty and depressive disorders
Source: Front Public Health. 2026 Mar 31;14:1788260. doi: 10.3389/fpubh.2026.1788260 (PMC13076108; doi:10.3389/fpubh.2026.1788260)
Supplement: Supplementary file 4 [file Table_4.docx]

Supplementary Material 4. Confirmatory factor analysis models and model fit comparison

Supplementary Figure 4A. One-factor and three factor CFA models


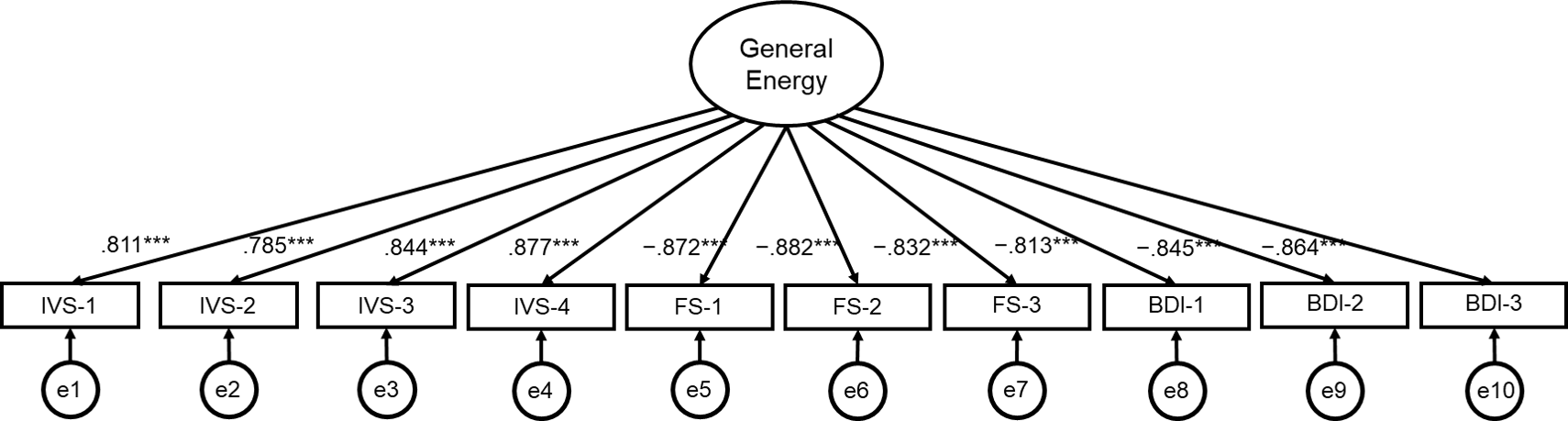


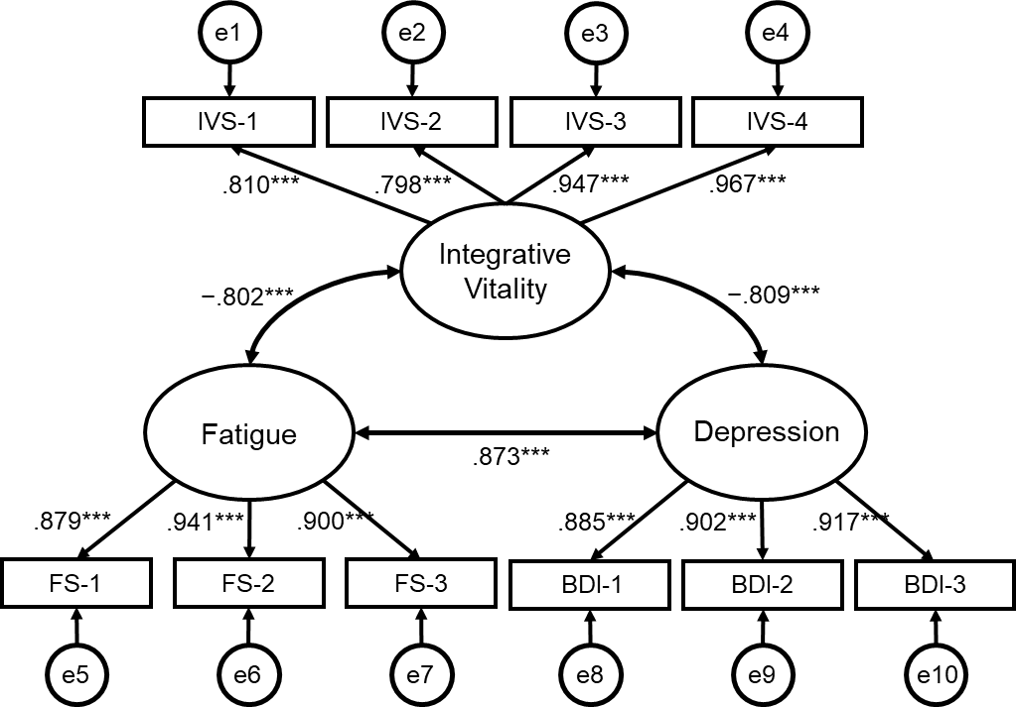


Supplementary Table 4B. Model fit indices for one-factor and three-factor measurement models

| Model | ***χ*^2^** | **df** | **CFI** | **TLI** | **RMSEA** | **SRMR** |
| --- | --- | --- | --- | --- | --- | --- |
| One-factor model | 318.970^***^ | 35 | 0.799 | 0.742 | 0.257 | 0.067 |
| Three-factor model | 133.250^***^ | 32 | 0.928 | 0.899 | 0.160 | 0.051 |
| *Note.* The chi-square difference test indicated that the three-factor model fit the data significantly better than the one-factor model (*Δχ*² = 185.72, *Δ*df = 3, *p* < .001).  ^***^ *p* < .001 | | | | | | |
